# Knockdown of NRMT enhances sensitivity of retinoblastoma cells to cisplatin through upregulation of the CENPA/Myc/Bcl2 axis

**DOI:** 10.1038/s41420-021-00622-w

**Published:** 2022-01-10

**Authors:** Zhongrui Li, Lan Zhang, Dongrui Liu, Zhanghui Yang, Di Xuan, Yi Zhang

**Affiliations:** 1 Department of Ophthalmology, The First Affiliated Hospital of Harbin Medical University, Harbin, 150001 P. R. China; 2 Department of Cardiology, the Fourth Affiliated Hospital of Harbin Medical University, Harbin, 150001 P. R. China

**Keywords:** Cancer, Diseases

## Abstract

Chemotherapy resistance of tumor cells causes failure in anti-tumor therapies. Recently, N-terminal regulator of chromatin condensation 1 methyltransferase (NRMT) is abnormally expressed in different cancers. Hence, we speculate that NRMT may pay a crucial role in the development of chemosensitivity in retinoblastoma. We characterized the upregulation of NRMT in the developed cisplatin (CDDP)-resistant retinoblastoma cell line relative to parental cells. Loss-of-function experiments demonstrated that NRMT silencing enhanced chemosensitivity of retinoblastoma cells to CDDP. Next, NRMT was identified to enrich histone-H3 lysine 4 trimethylation in the promoter of centromere protein A (CENPA) by chromatin immunoprecipitation assay. Rescue experiments suggested that CENPA reduced chemosensitivity by increasing the viability and proliferation and reducing apoptosis of CDDP-resistant retinoblastoma cells, which was reversed by NRMT. Subsequently, CENPA was witnessed to induce the transcription of Myc and to elevate the expression of B cell lymphoma-2. At last, in vivo experiments confirmed the promotive effect of NRMT knockdown on chemosensitivity of retinoblastoma cells to CDDP in tumor-bearing mice. Taken together, NRMT is an inhibitor of chemosensitivity in retinoblastoma. Those findings shed new light on NRMT-targeted therapies for retinoblastoma.

## Introduction

Retinoblastoma is known as one of the most common types of pediatric intraocular cancer [1]. Retinoblastoma is initiated by mutation of retinoblastoma gene alleles in a susceptible retinal cell. Uncontrolled cell division and recurrent genomic alterations in the process of tumorigenesis are caused by inactivation of tumor suppressor, retinoblastoma protein [2]. Systemic chemotherapy is proposed as the first-line treatment for retinoblastoma instead of radiation since the 1990s, and is effective in shrinking tumors while resulting in adverse effects such as hearing damage, risk of a second cancer along with long-term fertility issues [3]. Dismayingly, progression in therapies for this malignancy is largely restrained by poor public and medical awareness as well as insufficient rigorous clinical experiments for innovative treatments [4]. Cisplatin (CDDP), an DNA-damaging agent, is extensively applied as a chemotherapeutic drug [5]. However, chemotherapy resistance of tumor cells limits the decision of clinical therapies and hence emphasizes the importance of developing novel therapeutic approaches [6]. Fortunately, epigenetic deregulation of oncogenic pathways is responsible for tumorigenesis, and attention paid on this through whole-genome sequencing and epigenetic analyses may contribute to the identification of candidate genes as promising antineoplastic targets [7].

N-terminal regulator of chromatin condensation 1 methyltransferase (NRMT) is an eukaryotic methyltransferase capable of specifically methylating free α-amino proteins [8]. It was previously demonstrated that NRMT could methylate retinoblastoma protein whose mutation in susceptible retinal cells may give rise to the initiation of retinoblastoma [9]. NRMT, also known as NTMT1, is demonstrated as an oncogene in diverse human cancers such as melanoma, gastrointestinal and brain malignancies [10]. Centromere protein A (CENPA) is a centromere-specific histone-H3-like variant gene and its a-amino trimethylation by NRMT is an important event in the maintenance of centromere function and chromosome segregation [11, 12]. Myc is an aberrantly expressed transcription factor that acts as a crucial player in many biological processes such as proliferation and differentiation of cells, and participates in the pathogenesis of several cancers [13]. For instance, the abnormal expression of Myc was implicated in the progression of retinoblastoma and proposed as a promising therapeutic target for the treatment of this malignancy [14, 15] Moreover, a precious study demonstrated that CENPA hotspots could accumulate at the 8q24/Myc region and thus impact chromosome fragility of cancer cells [16]. B cell lymphoma-2 (Bcl2), an anti-apoptotic protein, was reported to facilitate cell proliferation and restrain apoptosis of retinoblastoma cells [17, 18]. Myc/Bcl2 co-expression was confirmed as vital prognostic factors in primary central nervous system diffuse large B cell lymphoma [19].

In light of those findings, it was speculated that NRMT may regulate the CENPA/Myc/Bcl2 axis to affect the chemoresistance in human cancers. Hence, the objective of the present study was to identify the regulatory potentials and mechanisms associated with NRMT to the develop strategy to minimize the chemoresistance in retinoblastoma cells.

## Results

### NRMT is highly expressed in retinoblastoma tissues and cells and promotes chemoresistance of retinoblastoma cells

**Fig. 1:**
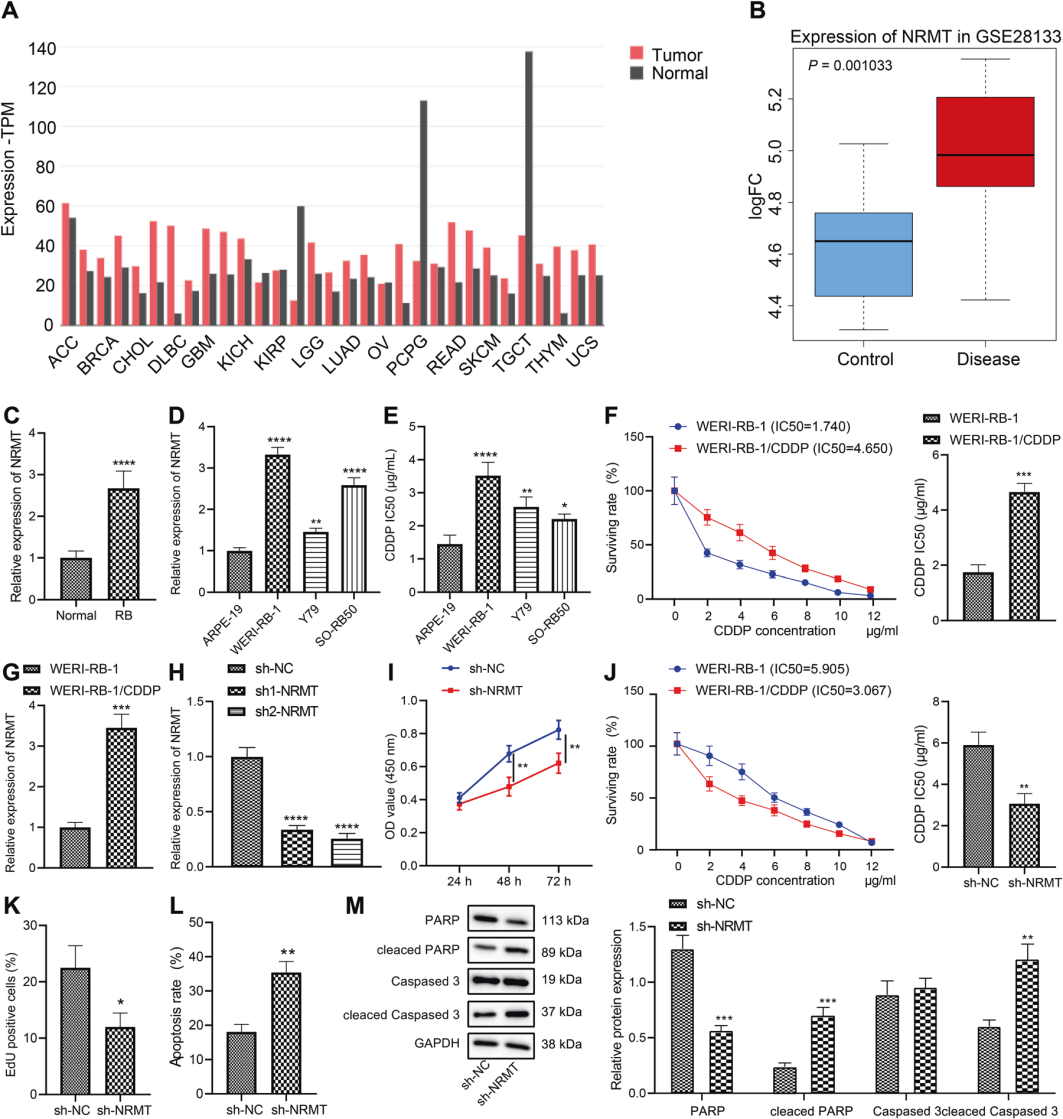
NRMT is highly expressed in retinoblastoma and inhibits chemosensitivity. **A** The expression of NRMT of 33 types of cancers in TCGA analyzed using GEPIA, T represents the expression of tumor tissues, N represents the expression of normal tissues, the upper horizontal axis represents various cancer abbreviations, red represents high expression in this cancer species, green represents low expression, and black indicates that the difference is not significant. **B** Box plot of NRMT expression in normal (blue box) and diseased retina (red box) in microarray dataset GSE28133. **C** The expression of NRMT in retinoblastoma and normal control tissues determined by RT-qPCR, *p < 0.05 vs. normal tissues. **D** The expression of NRMT in retinoblastoma (WERI-RB-1, Y79, and SO-RB50) and normal cell line ARPE-19 determined by RT-qPCR, *p < 0.05 vs. ARPE-19 cell line. **E** Sensitivity of WERI-RB-1 cells to CDDP shown by IC_50_ values after CDDP treatment assessed using CCK8. **F** IC_50_ values of WERI-RB-1 and WERI-RB-1/CDDP cell lines. **G** The expression of NRMT in WERI-RB-1 and WERI-RB-1/CDDP cell lines determined by RT-qPCR. **H** The expression of NRMT in WERI-RB-1/CDDP cells after transfection with sh-NRMT. **I** Cell viability after transfection with sh-NRMT assessed using CCK8 method. **J** IC_50_ values of cells after transfection with sh-NRMT measured by CCK8 assay. **K** Cell proliferation after transfection with sh-NRMT evaluated using EdU labeling assay. **L** Flow cytometric analysis of cell apoptosis after transfection with sh-NRMT. *p < 0.05 vs. cells transfected with sh-NC. **M** Expression of PARP, Cleaved PARP, caspase 3, and Cleaved caspase 3 in cells after transfection with sh-NRMT determined by western blot analysis. ^*/#^*p* < 0.05; ^**/##^*p* < 0.01; ^***/###^*p* < 0.001; ^****/####^*p* < 0.0001. The experiment was independently repeated three times.

According to the results of Gene Expression Profiling Interactive Analysis (GEPIA), NRMT (named NTMT1 in NCBI) was abnormally overexpressed in 19 (57.6%) of 33 cancers. NRMT was highly expressed in two neurological tumors, brain lower grade glioma and glioblastoma multiforme, and poorly expressed in 3 (9.1%) of 33 tumors, but not differentially expressed in the remaining 11 tumor types (Fig. 1A). The retinal detachment-related microarray GSE28133 was analyzed in the Gene Expression Omnibus (GEO) database, results of which determined that NRMT was highly expressed in the diseased retina (Fig. 1B).

To characterize the differential expression of NRMT in retinoblastoma tissues, tumor tissue and normal tissue samples were collected. The results of reverse transcription quantitative polymerase chain reaction (RT-qPCR) results showed that NRMT expression was elevated in the retinoblastoma tissues as compared to normal tissues (Fig. 1C). Consistently, relative to ARPE-19 cell line, NRMT was upregulated in human retinoblastoma cell lines WERI-RB-1, Y79, and SO-RB50 (Fig. 1D). The reduction of NRMT was more pronounced in the WERI-RB-1 cell line, which was therefore used for subsequent experiments.

To construct a CDDP-resistant cell line, the sensitivity of retinoblastoma cells to CDDP was evaluated by cell counting kit-8 (CCK8) assay. The results displayed that WERI-RB-1 cell line was more resistant to CDDP relative to the remaining retinoblastoma cell lines (Y79 and SO-RB50) (Fig. 1E). Then, we constructed a WERI-RB-1/CDDP-resistant cell line and detected the degree of resistance to CDDP in WERI-RB-1 and WERI-RB-1/CDDP cell lines. It was found that half maximal inhibitory concentration (IC_50_) value of the WERI-RB-1/CDDP cell line was significantly higher than that in the WERI-RB-1 cell line, suggesting the successful construction of a stable CDDP-resistant cell line (Fig. 1F).

To further confirm the association between NRMT and chemosensitivity of retinoblastoma, the expression of NRMT in WERI-RB-1 and WERI-RB-1/CDDP cells was determined by RT-qPCR. NRMT expression was higher in WERI-RB-1/CDDP cells relative to WERI-RB-1 cells (Fig. 1G). To examine the effects of NRMT on retinoblastoma cell resistance, NRMT was silenced in WERI-RB-1/CDDP cells and then the viability, IC_50_ values and apoptosis were assessed. As shown in Fig. 1H, a significant decrease in NRMT expression was induced by short hairpin RNA against NRMT (sh-NRMT) transfection, with sh-NRMT-2 presented a more notable decrease, so sh-NRMT-2 was selected for following experiments. Through the assessment of cell viability and IC_50_ values by CCK8 assay, it was found that knockdown of NRMT inhibited cell viability (Fig. 1I) and decreased its IC_50_ (Fig. 1J), hence promoting CDDP sensitivity of WERI-RB-1 cells. Similarly, 5-ethynyl-2′-deoxyuridine (EdU) labeling, and flow cytometric analyses suggested that silencing of NRMT inhibited proliferation (Figs. 1K and S1A), and enhanced apoptosis of WERI-RB-1/CDDP cells (Figs. 1L and S2A). The above results demonstrate that NRMT is overexpressed in retinoblastoma tissues and cells, while downregulation of NRMT inhibits CDDP resistance of retinoblastoma cells.

### NRMT represses chemosensitivity of retinoblastoma cells by promoting CENPA

**Fig. 2:**
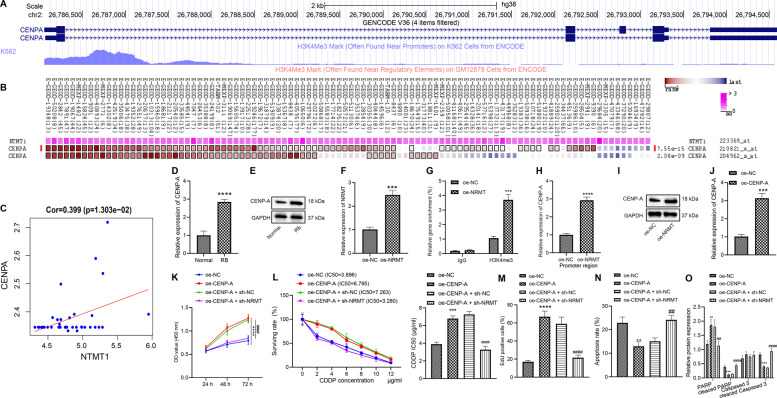
NRMT inhibits chemosensitivity of WERI-RB-1/CDDP cells by promoting CENPA expression. **A** The methylation modification sites of CENPA obtained by UCSC analysis. **B** Co-expression between NRMT and CENPA identified by MEM analysis. **C** The positive correlation between CENPA and NTMT identified by analysis of microarray dataset GSE28133. **D** The differential expression of CENPA in retinoblastoma determined by RT-qPCR. **E** The differential expression of CENPA in retinoblastoma determined by western blot analysis (*p < 0.05 vs. normal tissues). **F** The overexpression efficiency of NRMT determined by RT-qPCR. **G** H3K4me3 enrichment determined by ChIP assay. **H** The expression of CENPA measured by RT-qPCR. **I** The expression of CENPA measured by western blot analysis. **J** Overexpression efficiency of CENPA determined by RT-qPCR (*p < 0.05 vs. cells transfected with oe-NC). **K** Cell viability assessed by CCK8 assay. **L** IC_50_ value determined by CCK8 assay. **M** Cell proliferation assessed by EdU assay. **N** Cell apoptosis assessed by flow cytometry. **O** Expression of PARP, Cleaved PARP, caspase 3, and Cleaved caspase 3 in cells after transfection determined by western blot analysis. *p < 0.05 vs. cells transfected with oe-NC, ^#^*p* < 0.05 vs. cells transfected with oe-CENPA + sh-NC. ^*/#^*p* < 0.05; ^**/##^*p* < 0.01; ^***/###^*p* < 0.001; ^****/####^*p* < 0.0001. The experiment was independently repeated three times.

The methylation modification sites of CENPA were predicted using UCSC, and it was identified that histone-H3 lysine 4 trimethylation (H3K4me3) was highly enriched in CENPA promoter region (Fig. 2A). Co-expression analysis by MEM confirmed that NRMT and CENPA were highly co-expressed (Fig. 2B). Microarray GSE28133 was analyzed and NRMT expression was found to be notably positively correlated with CENPA expression (Fig. 2C). The results of RT-qPCR and western blot analysis revealed that CENPA expression was increased in the tumor tissues relative to that in normal tissues (Fig. 2D, E). To verify whether NRMT increased the expression of CENPA, the overexpression efficiency of plasmids harboring overexpressed NRMT (oe-NRMT) was determined using RT-qPCR, and results displayed that compared with that in cells transfected with negative control for overexpressed genes (oe-NC), NRMT expression was markedly increased in response to oe-NRMT (Fig. 2F). Next, the enrichment of H3K4me3 in the CENPA promoter region was measured by chromatin immunoprecipitation (ChIP) assay, retinoblastoma cells were transfected with oe-NC or oe-NRMT, and results demonstrated that the expression of H3K4me3 in CENPA promoter region increased remarkably in the cells transfected with oe-NRMT (Fig. 2G), indicating that CENPA could be regulated by NRMT and H3K4me3. RT-qPCR and western blot analysis illustrated that CENPA expression was increased in response to oe-NRMT (Fig. 2H, I). Above results suggested that NRMT could promote CENPA expression in retinoblastoma cells.

In order to detect the chemosensitivity of cells in response to CENPA overexpression, the overexpression efficiency of oe-CENPA was determined using RT-qPCR. The expression of CENPA was notably elevated in response to oe-CENPA (Fig. 2J). CCK8 assay found that overexpression of CENPA augmented viability of WERI-RB-1/CDDP cells (Fig. 2K) while increasing IC_50_ (Fig. 2L), suggesting that CENPA overexpression could inhibit the CDDP sensitivity of WERI-RB-1/CDDP cells. At the same time, the inhibition of NRMT restored the CDDP sensitivity inhibited by CENPA overexpression by decreasing the viability of WERI-RB-1/CDDP cells and IC_50_ value. Next, EdU assay indicated that overexpression of CENPA augmented the proliferation of cells, while inhibiting NRMT reversed the effect of CENPA on the proliferation of retinoblastoma cells (Figs. 2M and S1B). The results of flow cytometry (Figs. 2N and S2B) and western blot analysis (Figs. 2O and S3A) showed that overexpression of CENPA inhibited the apoptosis of cells, whereas NRMT silencing counteracted the effect of CENPA on the apoptosis of retinoblastoma cells. Above results showed that CENPA increased the viability of WERI-RB-1/CDDP cells and inhibited their CDDP sensitivity, while NRMT silencing could reverse the effect of CENPA on WERI-RB-1/CDDP cells.

### CENPA inhibits the chemosensitivity of retinoblastoma cells via activation of Myc

**Fig. 3:**
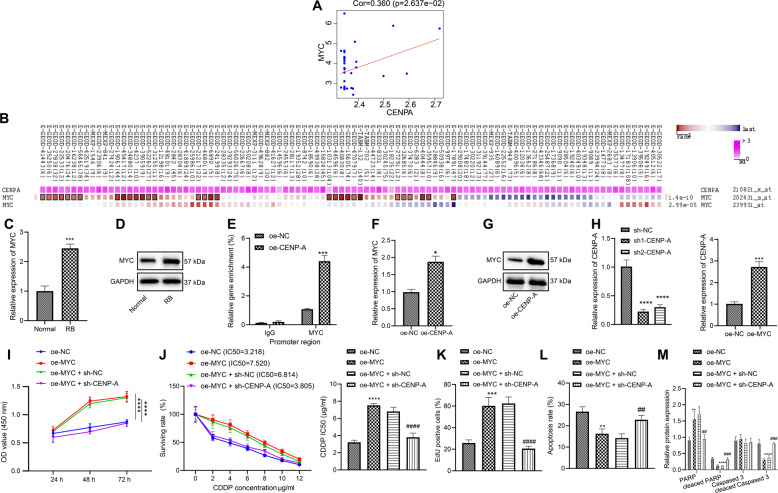
CENPA promotes the expression of Myc, and reverses the inhibitory effect of Myc on the chemosensitivity of WERI-RB-1/CDDP cells. **A** The positive correlation between CENPA and Myc identified through the analysis of microarray GSE28133. **B** The co-expression between CENPA and Myc identified by MEM analysis. **C** The differential expression of Myc in retinoblastoma determined by RT-qPCR. **D** The differential expression of Myc in retinoblastoma determined by western blot analysis (*p < 0.05 vs. normal tissues). **E** The binding of CENPA to Myc promoter region determined by ChIP assay. **F** The expression of Myc determined by RT-qPCR. **G** The expression of Myc determined by western blot analysis. **H** CENPA silencing and Myc overexpression efficiency determined by RT-qPCR (*p < 0.05 vs. cells transfected with oe-NC or sh-NC). **I** Cell viability assessed by CCK8 assay. **J** IC_50_ value determined by CCK8 assay. **K** Cell proliferation assessed by EdU assay. **L** Cell apoptosis assessed by flow cytometry. **M** Expression of PARP, Cleaved PARP, caspase 3, and Cleaved caspase 3 in cells after transfection determined by western blot analysis. *p < 0.05 vs. cells transfected with oe-NC; ^#^*p* < 0.05 vs. cells co-transfected with oe-Myc + sh-NC. ^*/#^*p* < 0.05; ^**/##^*p* < 0.01; ^***/###^*p* < 0.001; ^****/####^*p* < 0.0001. The experiment was independently repeated three times.

Analysis of the microarray dataset GSE28133 suggested the positive correlation between that CENPA and Myc (Fig. 3A), and analysis in MEM confirmed the co-expression of CENPA and Myc (Fig. 3B). Then, the differential expression of Myc in retinoblastoma was determined. According to the results of RT-qPCR and western blot analysis, relative to that in normal tissues, the expression of Myc was increased in the retinoblastoma tissues (Fig. 3C, D). To verify whether CENPA binds to the Myc promoter region, ChIP assay was conducted to detect the CENPA enrichment in the Myc promoter region, the results illustrated that the enrichment of CENPA in the Myc promoter region increased markedly in response to oe-CENPA (Fig. 3E), indicating that Myc could be regulated by CENPA. Next, RT-qPCR and western blot analysis showed that Myc expression increased in response to oe-CENPA (Fig. 3F, G). Hence, CENPA augmented the expression of Myc in WERI-RB-1/CDDP cells.

In order to investigate whether CENPA activated Myc through transcription and affected the chemosensitivity, sh-CENPA was used to silence CENPA expression in WERI-RB-1/CDDP cells. RT-qPCR analysis found that compared with that in cells transfected with sh-NC, the expression of CENPA decreased in cells transfected with sh-CENPA-1 or sh-CENPA-2, and the decrease was more notable in the presence of sh-CENPA-1, which was therefore selected as the following experimental sequence. Besides, expression of Myc was remarkably increased in response to oe-Myc (Fig. 3H). Then, CCK8 assay found that overexpression of Myc augmented cell viability (Fig. 3I), while increasing IC_50_ (Fig. 3J), suggesting that Myc overexpression could inhibit the CDDP sensitivity of WERI-RB-1/CDDP cells. However, downregulation of CENPA negated the effects of Myc overexpression on the viability and IC_50_ value of WERI-RB-1/CDDP cells. Next, the results of EdU (Figs. 3K and S1C) and flow cytometry (Figs. 3L and S2C) exhibited that overexpression of Myc augmented the proliferation of WERI-RB-1/CDDP cells and suppressed their apoptosis, while silencing of CENPA reversed the effect of Myc on the proliferation and apoptosis. Further western blot analysis also provided consistent evidence (Figs. 3M and S3B). In short, overexpression of Myc augmented the proliferation of WERI-RB-1/CDDP cells and inhibited their CDDP sensitivity while silencing of CENPA can reverse the effect of Myc on WERI-RB-1/CDDP cells.

### Myc inhibits the chemosensitivity of retinoblastoma cells via upregulation of Bcl2

**Fig. 4:**
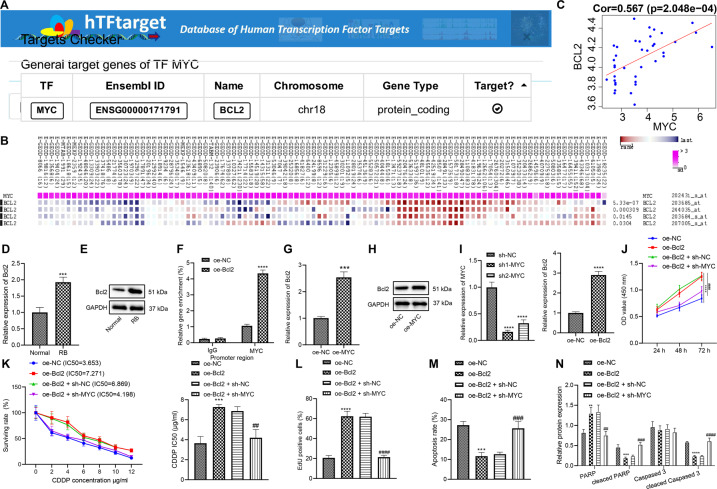
Myc promotes Bcl2 expression to inhibit chemosensitivity of WERI-RB-1/CDDP cells. **A** The relationship between Myc and Bcl2 identified by hTFtarget. **B** Co-expression of Myc and Bcl2 explored by MEM analysis. **C** The positive correlation between Myc and Bcl2 certified by the analysis of microarray GSE28133. **D** Differential expression of Bcl2 in retinoblastoma determined by RT-qPCR. **E** Differential expression of Bcl2 in retinoblastoma determined by western blot analysis (*p < 0.05 vs. normal tissues). **F** The binding of Myc to the Bcl2 promoter binding detected by ChIP assay. **G** Bcl2 expression determined by RT-qPCR. **H** Bcl2 expression determined by western blot analysis. **I** Myc silencing and Bcl2 overexpression efficiency determined by RT-qPCR (*p < 0.05 vs. cells transfected with oe-NC or sh-NC). **J** Cell viability assessed by CCK8 assay. **K** IC_50_ value determined by CCK8 assay. **L** Cell proliferation assessed by EdU assay. **M** Cell apoptosis assessed by flow cytometry. **N** Expression of PARP, Cleaved PARP, caspase 3, and Cleaved caspase 3 in cells after transfection determined by western blot analysis. *p < 0.05 vs. cells transfected with oe-NC; ^#^*p* < 0.05 vs. cells co-transfected with oe-Bcl2 + sh-NC. ^*/#^*p* < 0.05; ^**/##^*p* < 0.01; ^***/###^*p* < 0.001; ^****/####^*p* < 0.0001. The experiment was independently repeated three times.

The hTFtarget analysis suggested that Myc can regulate Bcl2 (Fig. 4A), and MEM analysis displayed the co-expression of Myc and Bcl2 (Fig. 4B). In microarray GSE28133, Myc and Bcl2 expression were positively correlated (Fig. 4C). The results of RT-qPCR and western blot analysis illustrated that Bcl2 expression increased in the retinoblastoma tissues compared with that in the normal tissues (Fig. 4D, E). To verify the binding of Myc to the promoter region of Bcl2, the enrichment of Myc in the Bcl2 promoter region was determined by ChIP. It was established that the enrichment of Myc in the Bcl2 promoter region in response to the oe-Myc was increased remarkably (Fig. 4F), indicating that Bcl2 could be regulated by Myc. The results of RT-qPCR and western blot analysis supported this finding that Bcl2 expression was increased in response to oe-Myc (Fig. 4G, H). In a word, Myc could upregulate Bcl2 expression in retinoblastoma cells.

In order to detect whether Myc activated Bcl2 through transcription and affected the chemosensitivity of cells, Myc was knocked down using sh-Myc in WERI-RB-1/CDDP cells. RT-qPCR validated that the expression of Myc was decreased in cells transfected with sh-Myc-1 or sh-Myc-2, and it was decreased more notably in response to sh-Myc-1, so sh-Myc-1 was selected as the subsequent experimental sequence (Fig. 4I). Moreover, the expression of Bcl2 was increased notably in response to oe-Bcl2. The results of CCK8, EdU, flow cytometry, and western blot analysis (Figs. 4N and S3C) showed that overexpression of Bcl2 augmented cell viability (Fig. 4J), increased IC_50_ value in WERI-RB-1/CDDP cells (Fig. 4K), and enhanced their proliferation (Figs. 4L and S1D) but repressed apoptosis (Figs. 4M and S2D). However, inhibition of Myc neutralized the effect of Bcl2 on those functions of WERI-RB-1/CDDP cells. Hence, Myc impeded the chemosensitivity of retinoblastoma cells by upregulating Bcl2.

### NRMT represses chemosensitivity of retinoblastoma cells by upregulating Bcl2

**Fig. 5:**
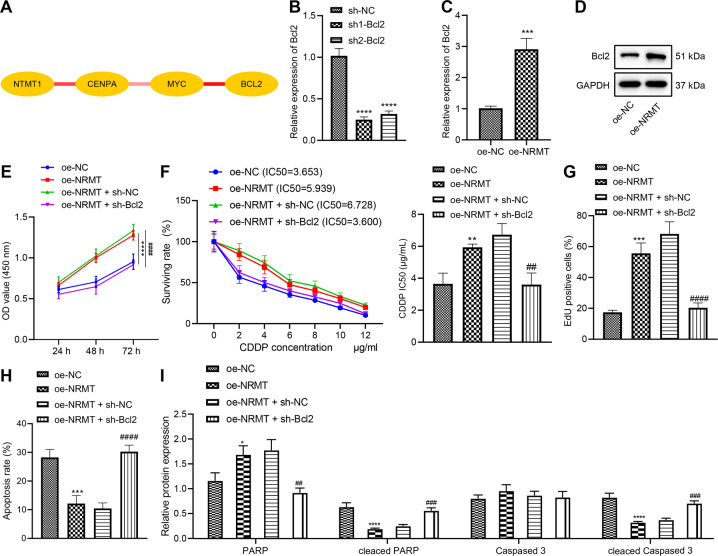
NRMT inhibits chemosensitivity of WERI-RB-1/CDDP cells through Bcl2. **A** The interaction among NRMT, CENPA, Myc, and Bcl2 predicted by String. The redder the line between genes, the stronger the interaction, and the whiter the weaker. **B** The silencing efficiency of Bcl2 determined by RT-qPCR (*p < 0.05 vs. cells transfected with sh-NC). **C** The expression of Bcl2 determined by RT-qPCR. **D** The expression of Bcl2 determined by western blot analysis (*p < 0.05 vs. cells transfected with oe-NC). **E** Cell viability assessed by CCK8 assay. **F** IC_50_ value determined by CCK8 assay. **G** Cell proliferation assessed by EdU assay. **H** Cell apoptosis assessed by flow cytometry. **I** Expression of PARP, Cleaved PARP, caspase 3, and Cleaved caspase 3 in cells after transfection determined by western blot analysis. *p < 0.05 *vs*. cells transfected with oe-NC; ^#^*p* < 0.05 vs. cells co-transfected with oe-NRMT + sh-NC. *^/#^*p* < 0.05; **^/##^*p* < 0.01; ***^/###^*p* < 0.001; ****^/####^*p* < 0.0001. The experiment was independently repeated three times.

Above-mentioned results have supported that Myc promoted Bcl2 expression in retinoblastoma cells resistant to CDDP. The interaction relationship among NRMT, CENPA, Myc, and Bcl2 was analyzed through the String method. We found a high interaction score among NRMT-CENPA, CENPA-Myc, and Myc-Bcl2 (Fig. 5A). RT-qPCR determined that the expression of Bcl2 was reduced in cells transfected with sh-Bcl2-1 or sh-Bcl2-2, and cells transfected with sh-Bcl2-1 presented a more obvious downregulation, so sh-Bcl2-1 was selected for subsequent experiments (Fig. 5B). The results of RT-qPCR and western blot analysis demonstrated that Bcl2 expression in response to oe-NRMT was elevated notably (Fig. 5C, D). The results of CCK8, EdU, and flow cytometry revealed that overexpression of NRMT facilitated cell viability (Fig. 5E), elevated IC_50_ value (Fig. 5F), and enhanced cell proliferation (Figs. 5G and S1E) but repressed apoptosis (Figs. 5H and S2E) of WERI-RB-1/CDDP cells. Western blot analysis provided experimental data for verification purpose (Figs. 5I and S3D). However, simultaneous suppression of Bcl2 could partially reverse the pro-proliferative and anti-apoptotic effects of NRMT on WERI-RB-1/CDDP cells and restore their sensitivity to CDDP. To sum up, overexpression of NRMT suppressed chemosensitivity of WERI-RB-1/CDDP cells by enhancing Bcl2.

### NRMT knockdown enhances chemosensitivity of retinoblastoma tissues and represses tumor growth in vivo

In vitro experiments have verified that NRMT inhibits chemosensitivity of retinoblastoma cells through the CENPA/Myc/Bcl2 axis, and in vivo experiments were designed to verify whether NRMT could inhibit chemosensitivity of retinoblastoma tissues through the CENPA/Myc/Bcl2 axis to promote tumorigenesis. On the 20th day after retinoblastoma cell inoculation, the sensitivity of retinoblastoma cells to CDDP was assessed after overexpression of NRMT. The tumor-bearing mice were intraperitoneally injected with CDDP and phosphate buffer saline (PBS, as a control) once the tumor volume over 100 mm^3^. Treatment was terminated on the 28th day and tumor tissues were collected for subsequent experiments.

**Fig. 6:**
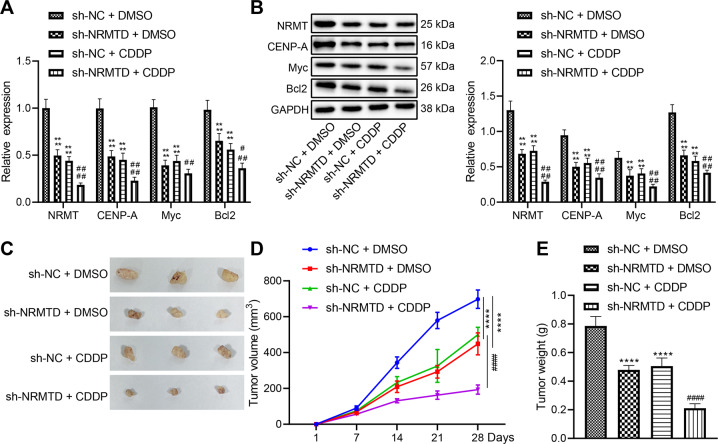
NRMT acted as an oncogene in retinoblastoma by inhibiting chemosensitivity in vivo. **A** The expression of CENPA, Myc, and Bcl2 determined by RT-qPCR. **B** The expression of CENPA, Myc, and Bcl2 determined by western blot analysis. **C** Representative images of xenografted tumors. **D** The statistical graph of tumor volume. **E** The statistical graph of tumor weight. *p < 0.05 vs. mice infected with sh-NC + PBS, ^#^*p* < 0.05 vs. that of mice infected with sh-NC + CDDP. *^/^^#^*p* < 0.05; **^/##^*p* < 0.01; ***^/###^*p* < 0.001; ****^/####^*p* < 0.0001. The experiment was independently repeated three times.

RT-qPCR and western blot analysis suggested that the expression of CENPA, Myc, and Bcl2 was reduced in response to NRMT knockdown. The expression of CENPA, Myc, and Bcl2 was significantly reduced after CDDP treatment, and this reduction was enhanced after NRMT silencing (Fig. 6A, B). The xenografted tumor volume and weight were notably reduced in nude mice in response to NRMT knockdown. CDDP treatment also inhibited tumor growth and this inhibitory effect was enhanced by NRMT downregulation (Fig. 6C–E). The aforementioned results demonstrated that NRMT silencing could inhibit the expression of CENPA, Myc, and Bcl2, and inhibited tumor growth in vivo by enhancing chemosensitivity.

## Discussion

Retinoblastoma is the most common pediatric intraocular tumor that seriously impairs the vision and health of infants and young children [20]. Due to difficulty in early diagnosis of retinoblastoma, tumor metastasis and relapse cause big challenges in the treatment [21]. In this study, NRMT was determined to be highly expressed in CDDP-resistant retinoblastoma cells. NRMT was previously demonstrated to present a notable upregulation in numerous cancers and knockdown of NRMT may serve as anticancer therapeutics by inducing cell mitotic defects [10]. Hence, our study focused on the possible tumor-supporting effects of NRMT on retinoblastoma cells, and our results suggested an accelerative effect of NRMT knockdown on the chemosensitivity both in vitro and in vivo. Here, we also identified a CENPA/Myc/Bcl2 axis mediated by NRMT underlying its effect.

The first general observation of our study was that NRMT downregulation led to enhanced chemosensitivity of retinoblastoma cells. Consistent with the elevation in the retinoblastoma tissues and cells, NRMT expression was also increased in patients with gastrointestinal cancer relative to healthy controls [22]. Also, it has been reported that loss of NRMT may enhance the sensitivity of tumors to varied DNA-damaging chemotherapeutics [23]. Similarly, DNA methyltransferase 1 is also known as a methyltransferase to contribute to the pathogenesis of retinoblastoma [24]. In addition, a more recent study revealed that the repression of histone methyltransferase recruitment exerted an inhibiting function on retinoblastoma development, and thus related to a more favorable therapeutic effect [25]. In short, NRMT silencing served as a promoter of chemosensitivity of retinoblastoma cells.

Subsequently, our study evidenced that NRMT repressed the chemosensitivity of retinoblastoma cells by elevating the expression of CENPA. CENPA is highly expressed in human cancer genome and also identified as a regulator of RB protein [16, 26]. Another study has demonstrated that the trimethylation of CENPA can be augmented by NRMT and thus further enhance mitosis, centromere function, and chromosomes segregation [11]. Then, CENPA was observed to increase the expression of Myc through transcriptional activation in the current study. Myc is a protooncogene regulating an array of cellular processes such as proliferation and metabolism whose overexpression triggers various malignancies [27]. For instance, Myc upregulation acted as an enhancer of the viability, migration, and invasion of retinoblastoma cells [28]. Similar to our finding, a previous study has also affirmed that CENPA is responsible for the amplification and overexpression of Myc, and further results in histone variant mislocalization of human cancer cells [29]. Further, our study demonstrated that Myc elevated the expression of the anti-apoptotic protein Bcl2. Bcl2 is critical for cell death processes and is proposed as a promising target for human gene therapy in cancers [30]. Bcl2 was illustrated to prevent apoptosis of retinoblastoma cells [17, 18]. Additionally, another study revealed that the expression of Bcl2 was negatively correlated with the chemoradioresistance of mesenchymal lung cancer cells [31]. More importantly, prior research has illustrated that Bcl2 and Myc are co-expressed in diffuse large B cell lymphoma cells and their higher expression is accountable for the poorer outcome of cancer [32]. Our experiments demonstrated that NRMT overexpression could suppress chemosensitivity of WERI-RB-1/CDDP cells by upregulating Bcl2. At last, our in vivo experimental data confirmed the downregulation of CENPA/Myc/Bcl2 by NRMT silencing, which possibly could be the mechanism responsible for the enhanced chemosensitivity of retinoblastoma cells.

**Fig. 7:**
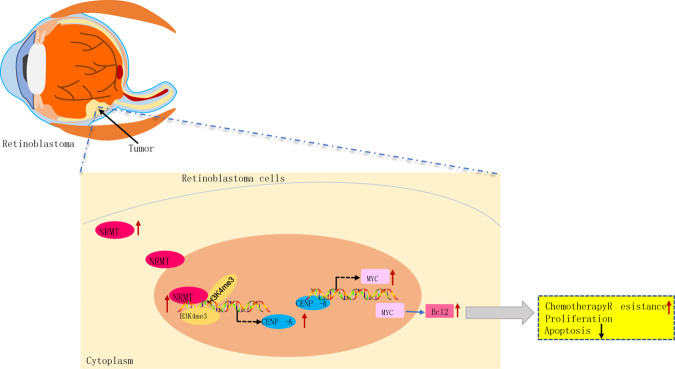
Schematic diagram illustrating the action of NRMT on chemosensitivity of retinoblastoma cells via the CENPA-mediated Myc/Bcl2 axis. NRMT upregulates CENPA through histone methylation to promote the enrichment of CENPA on the Myc promoter region, by which Bcl2 expression is elevated so that chemosensitivity of retinoblastoma cells is suppressed.

In summary, our study identifies a regulatory axis NRMT/CENPA/Myc/Bcl2 implicated in the resistance to CDDP in retinoblastoma cells, which contributes to the comprehensive understanding of molecules and pathways that control CDDP-induced apoptosis (Fig. 7). NRMT knockdown is demonstrated to enhance the chemosensitivity of retinoblastoma cells to CDDP; this aspect of the present study could potentially pave the way for future studies to explore novel therapeutic targets in preventing chemoresistance.

## Materials/subjects and methods

### Ethics statements

All experiments involving human beings were approved by the Ethics Committee of the First Affiliated Hospital of Harbin Medical University and conformed to the principles of Declaration of Helsinki. Informed consent documentation was obtained from the legal guardian of each patient prior to experiments. The experimental procedures involving animals are in line with the guidelines for the care and use of laboratory animals originating in the National Institutes of Health.

### Bioinformatics analysis

Novel key genes and downstream pathways of retinoblastoma were predicted by searching existing literature, the data from transcriptomic analysis of human retinal detachment (GSE28133) were obtained from GEO database (https://www.ncbi.nlm.nih.gov/gds), and the expression of key genes in retinal diseases was analyzed by combining with the joint data of various cancer obtained from GEPIA (http://gepia2.cancer-pku.cn/#index), TCGA (https://portal.gdc.cancer.gov/), and GTEx (https://www.gtexportal.org/home/index.html). By analyzing the correlation of gene expression in microarray GSE28133 and gene co-expression in MEM (https://biit.cs.ut.ee/mem/index.cgi), the downstream pathways were screened. USCS (http://genome.ucsc.edu/) was used to obtain the methylation binding site. The relationship between transcription factors and downstream genes was determined through hTFtarget (http://bioinfo.life.hust.edu.cn/hTFtarget#!/), and the interaction relationship between genes was analyzed using String (https://string-db.org/). Correlation analysis and expression difference analysis are conducted using R language, and interaction diagrams are drawn through Cytoscape (https://cytoscape.org/).

### Study subjects

Tumor tissues were collected from 2 patients with retinoblastoma, while normal tissue samples were from 5 patients who suffered from ophthalmorrhexis at the First Affiliated Hospital of Harbin Medical University between July 2014 and July 2018. None of the enrolled patients had received anti-tumor therapy before surgery. Patients were pathologically diagnosed with retinoblastoma by independent film reading of three pathologists [33]. Patients conformed to the following conditions were excluded, including patients who had co-morbid severe malnutrition, other tumors or cardiopulmonary dysfunction, or those who received medications such as immunosuppressive agents, hormones drugs or blood products, pre-operative radiotherapy, immunotherapy or other related therapies.

### Cell culture

Retinoblastoma cell lines WERI-RB-1 (HTB-169) and Y79 (HTB-18), and human retinal microvascular endothelial cell (hRMEC) ARPE-19 (CRL-2302) were purchased from the American Type Culture Collection (ATCC, USA, https://www.atcc.org/), while retinoblastoma cell line SO-RB50 (QCB1533) was purchased from QCHENG Biotech Co., Ltd (Shanghai, China). The cells were cultured using Roswell Park Memorial Institute (RPMI) 1640 supplemented with 10% fetal bovine serum (FBS), 100 U/mL penicillin, and 100 U/mL streptomycin in an incubator at 37 °C with 5% CO_2_. Cells were detached with 0.25% trypsin and passaged in a ratio of 1:3. The cells were seeded in six-well plates at a density of 3 × 10^5^ cells/well. When the cell confluence reached 70–80%, the cell line with the highest NRMT expression was screened for subsequent experiments using RT-qPCR.

### Construction of CDDP-resistant cell line

After routine recovery, the human retinoblastoma cell line was cultured in complete RPMI 1640 medium containing 10% FBS, 100 kU/L penicillin, and 100 mg/L streptomycin in an incubator at 37 °C with 5% CO_2_. The CDDP-resistant retinoblastoma cell line WERI-RB-1/CDDP was induced by exposure to gradually increased concentration of CDDP (1, 2, 3, 4, and 5 mg/L). The medium was renewed with CDDP-free medium every 24 h after treatment, and then passaged in the stable growth phase. This step was repeated five times and then the CDDP concentration was altered to continue the culture. The above steps were repeated three times for each concentration. After a total of 190 days of induction, WERI-RB-1 cells were stably grown and normally passaged in medium containing 5 mg/L CDDP. The WERI-RB-1/CDDP cells were successfully induced.

### Cell transfection

The cells were cultured in Dulbecco’s modified Eagle’s medium (Invitrogen, Carlsbad, CA) supplemented with 7% FBS (Gibco, Carlsbad, CA) and 10 μg/mL streptomycin in an incubator at 37 °C with 5% CO_2_. The cells in the logarithmic phase were trypsinized and seeded in a six-well plate at a concentration of 1 × 10^5^ per well. After 24 h of culture, the cell confluence reached 75%, and the cells were transfected according to specifications of Lipofectamine 2000 kit (Invitrogen) for 48 h. The plasmids and sequences were purchased from GenePharma (Shanghai, China).

### RNA isolation and quantification

Tissue total RNA was extracted using TRIzol reagent (16096020, Thermo Fisher Scientific, Waltham, MA), 5 μg of which was reversely transcribed into cDNA according to the cDNA synthesis kit (K1622; Fermentas International Inc., Ontario, Canada), using cDNA as a template, with U6 as internal controls. Then, real-time PCR was performed according to the instructions of the TaqMan Gene Expression Assays protocol (Applied Biosystems, Foster City, CA) to determine gene expression level, with glyceraldehyde-3-phosphate dehydrogenase (GAPDH) used as an internal reference. Primer sequences are shown in Supplementary Table 1. The relative expression was calculated using 2^−^^ΔΔCt^ method.

### Western blot assay

The tissue and cellular proteins were isolated using radioimmunoprecipitation assay lysis buffer (BB-3209, BestBio, Shanghai, China). The supernatant was collected after high-speed centrifugation and the protein concentration was determined using a bicinchoninic acid assay kit (Thermo Fisher Scientific). After protein separation by sodium dodecyl sulfate polyacrylamide gel electrophoresis, the proteins were transferred onto a polyvinylidene fluoride membrane at a constant voltage of 80 V. After blocked for 1 h, the membrane was incubated overnight at 4 °C with primary antibodies: rabbit anti-human NRMT (ab72660, 1:500, Abcam, Cambridge, UK), CENPA (ab13939, 1:1000, Abcam), Myc (ab32, 1:1000, Abcam), Bcl2 (ab185002, 1:1000, Abcam), poly-ADP-ribose polymerase (PARP) (556494, 1:1000, BD-Pharmingen, San Diego, CA), caspase 3 (ab32351, 1:1000, Abcam), and Cleaved caspase 3 (ab32042, 1:1000, Abcam). The goat anti-rabbit immunoglobulin (IgG) (ab205718, 1:10,000, Abcam) as the secondary antibody was added for 1-h incubation at 37 °C. The membrane was developed by enhanced chemiluminescence and photographed by SmartView Pro 2000 (UVCI-2100, Major Science, Saratoga, CA). The band intensity was quantified using Quantity One software. Each set of experiments was repeated three times.

### ChIP

To study the enrichment of H3K4me3 in the promoter region of the CENPA, ChIP was performed using EZ-Magna ChIP A/G Kit (17-371, Millipore, Billerica, MA) according to the manufacturer’s instructions. After the cells were sonicated, centrifugation was performed at 4 °C at the speed of 12,000 × *g* for 10 min to remove the undissolved pellet. The harvested supernatant was incubated with Protein G Agarose at 4 °C for 1 h, and then centrifuged at 5000 × *g* for 1 min. Ten microliters (1%) collected supernatant was used as “Input” and the remaining supernatant was incubated with antibody to H3K4me3 (ab185637, 1:20, Abcam) or NC rabbit anti-human IgG (ab2410, 1:25, Abcam) at 4 °C overnight. The protein–DNA complex was incubated with Protein G Agarose at 4 °C for 1 h. After centrifugation at 5000 × *g* for 1 min, the supernatant was discarded, and the protein–DNA complex was eluted. The cross-linking was reversed at 65 °C overnight, and the DNA fragment was purified and harvested for RT-qPCR.

To study the enrichment of CENPA in the promoter region of Myc and the enrichment of Myc in the promoter region of Bcl2, cells in the logarithmic growth phase were subjected to the above-mentioned procedures (part of the DNA fragments were taken as Input). The collected supernatant was settled into three tubes, and incubated with NC anti-mouse IgG (ab2410, 1:25, Abcam) and the specific antibodies to CENPA (ab13939, 1:50, Abcam), and Myc (ab32, 1:50, Abcam) at 4 °C overnight. Endogenous DNA–protein complexes were precipitated using Protein Agarose/Sepharose, the supernatant was aspirated after brief centrifugation, and the non-specific complexes were rinsed. The cross-linking was reversed at 65 °C overnight, and the DNA fragments were purified and recovered using phenol/chloroform. Input was taken as internal reference to detect the binding of specific proteins in the promoter regions of Myc and Bcl2.

### EdU labeling

The cells in the logarithmic phase were seeded into 24-well plates, with three replicate wells in each group. Next, the cells were incubated with EdU (C10341-1, RiboBio) at a final concentration of 10 μmol/L for 2 h. The cells were fixed in PBS containing 4% paraformaldehyde for 15 min. Subsequently, the cells were permeabilized with 0.5% Triton-100 in PBS for 20 min. After incubation with 100 μL of Apollo® 567 (RiboBio) for 30 min in the dark, the cells were stained with 1× Hoechst 33342 for 30 min. Cells were rinsed twice with PBS containing 3% bovine serum albumin after each reaction. The number of positive cells (red) was counted under a fluorescence microscope (FM-600, Shanghai Pudan Optical Chemical Instrument Co., Ltd, Shanghai, China).

### CCK8

When the cell confluence reached about 80%, the cells were detached with 0.25% trypsin and dispersed into single-cell suspension. The 3 × 10^3^–6 × 10^3^ cells were seeded into a 96-well plate at a volume of 200 μL per well, with six parallel wells. At 24, 48, and 72 h post-culture, the cells were incubated with 10 μL of CCK8 (VP757, Dojindo, Kumamoto, Japan) per well for 2 h. The optical density (OD) value was calculated using an enzyme-linked immunosorbent assay (BIOBASE-EL10A, Jinan Boxin Biotechnology Co., Ltd, Jinan, China) and each well was read at the excitation wavelength of 450 nm. The cell viability curve was plotted with the time point as the *X*-axis and the OD value as the *Y*-axis. IC_50_, the concentration of cells with a survival rate of 50%, was calculated according to log values by Graphpad Prism.

### Flow cytometry

After transfection for 48 h, the transfected cells were treated with 25 μg/mL for 24 h. The apoptosis of retinoblastoma cells was assessed using Annexin V-fluorescein isothiocyanate (FITC)/propidium iodide (PI) double staining kit (556547; Shanghai SOJIA Biotechnology Co., Ltd, Shanghai, China). The cells were stained with 5 μL of Annexin V-FITC for 15 min and 5 μL of PI for 5 min. The fluorescence was detected on a flow cytometer (Cube6, Partec, Germany) at the excitation wavelength of 480 nm, while FITC fluorescence at 575 nm and PI fluorescence were detected at 530 nm.

### Tumor formation in nude mice

The stably transfected cells were resuspended in serum-free RPMI 1640 medium (Gibco) to cell suspension (1 × 10^7^ cells/mL). In total, 32 specific pathogen-free nude mice aged 4–6 weeks and weighing 18–20 g were purchased from Shanghai SLAC Laboratory Animal Co., Ltd (Shanghai, China) and assigned into four groups on a random basis with eight mice in each group. The cells were treated with 1 mL 0.25% trypsin and cultured in an incubator at 37 °C. The cell pellet was collected after centrifugation and then pipetted into single-cell suspension (2 × 10^6^ cells). The cells were resuspended in 50 μL normal saline and then mixed with 50 μL Matrigel Matrix (1:1). The mixture was inoculated into the armpit of nude mice. When tumor volume reached 100 mm^3^, nude mice were administrated with CDDP (2 mg/kg) through intraperitoneal injection one time every 2 days for 2 weeks. After anesthesia with 3% pentobarbital sodium (1 mL/100 g; P3761, Sigma-Aldrich, St. Louis, MO), mice were disinfected and euthanized after 28 days to collect tumors.

### Statistical analysis

The data were presented as mean ± standard deviation, as processed using SPSS 21.0 (IBM Corp., Armonk, NY). Differences in values were defined as significant if *p* ≤ 0.05. Statistical analysis included independent sample *t*-test for two-group comparison or one-way analysis of variance (ANOVA) with the Tukey’s post hoc test for multi-group comparisons. Cell viability or tumor volume at different time points was compared using two-way or repeated measures ANOVA, followed by Bonferroni post hoc test.

## Supplementary information

All supplementary information

## Data Availability

The data that support the findings of this study are available from the corresponding author upon reasonable request.
