# Supplementary material for: Knockdown of NRMT enhances sensitivity of retinoblastoma cells to cisplatin through upregulation of the CENPA/Myc/Bcl2 axis
Source: Cell Death Discov. 2022 Jan 10;8:14. doi: 10.1038/s41420-021-00622-w (PMC8748520; doi:10.1038/s41420-021-00622-w)
Supplement: Supplementary file 1 — All supplementary information [file 41420_2021_622_MOESM1_ESM.docx]

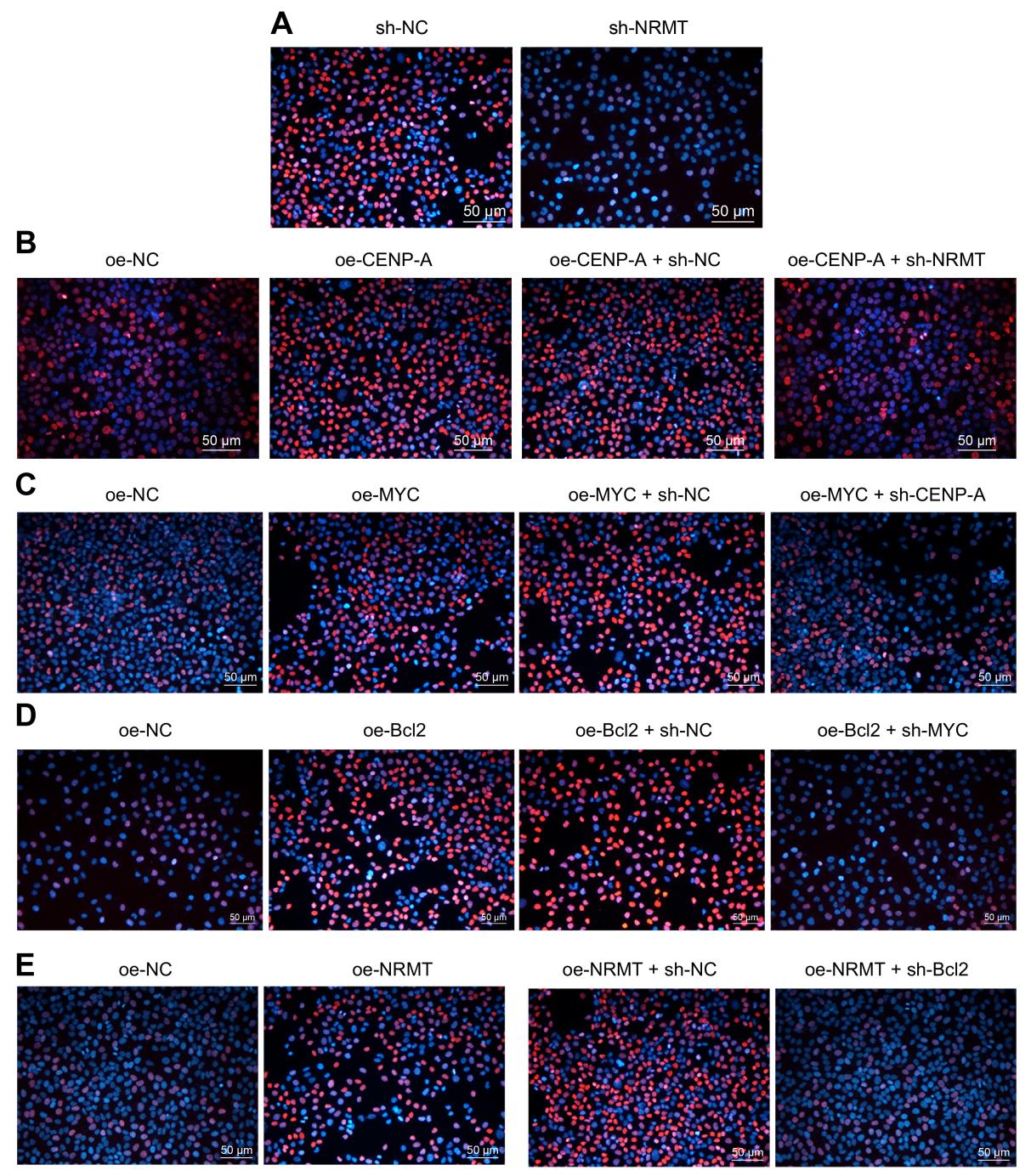


**Fig. S1** Representative images of EdU assay. A, Cell proliferation in presence of sh-NRMT assessed by EdU assay. B, Cell proliferation in presence of oe-CENPA or oe-CENPA + sh-NRMT assessed by EdU assay. C, Cell proliferation in presence of oe-Myc or oe-Myc + sh-CENPA assessed by EdU assay. D, Cell proliferation in presence of oe-Bcl2 or oe-Bcl2 + sh-Myc assessed by EdU assay. E, Cell proliferation in presence of oe-NRMT or oe-NRMT + sh-Bcl2 assessed by EdU assay.


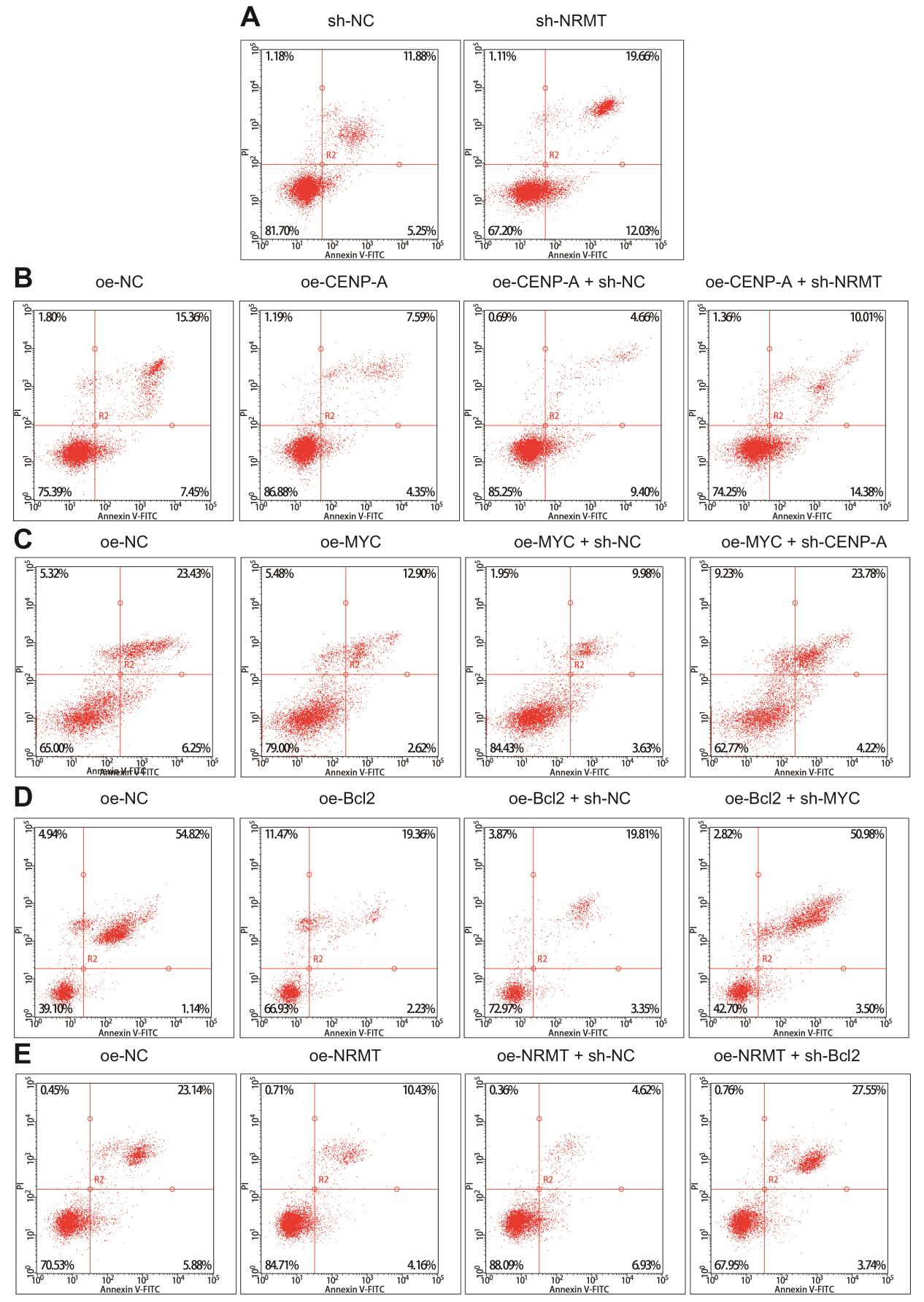


**Fig. S2** Representative images of flow cytometry. A, Cell apoptosis in presence of sh-NRMT assessed by flow cytometry. B, Cell apoptosis in presence of oe-CENPA or oe-CENPA + sh-NRMT assessed by flow cytometry. C, Cell apoptosis in presence of oe-Myc or oe-Myc + sh-CENPA assessed by flow cytometry. D, Cell apoptosis in presence of oe-Bcl2 or oe-Bcl2 + sh-Myc assessed by flow cytometry. E, Cell apoptosis in presence of oe-NRMT or oe-NRMT + sh-Bcl2 assessed by flow cytometry.


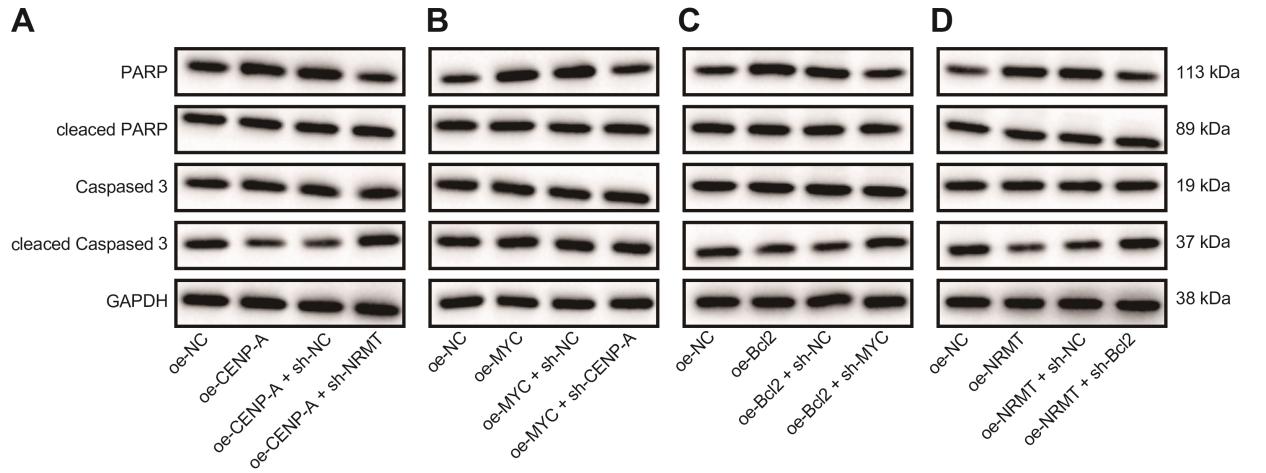


**Fig. S3** Representative images of Western blots. A, Expression of PARP, Cleaved PARP, caspase 3 and Cleaved caspase 3 in cells after transfection of oe-CENPA or oe-CENPA + sh-NRMT determined by Western blot analysis. B, Expression of PARP, Cleaved PARP, caspase 3 and Cleaved caspase 3 in cells after transfection of oe-Myc or oe-Myc + sh-CENPA determined by Western blot analysis. C, Expression of PARP, Cleaved PARP, caspase 3 and Cleaved caspase 3 in cells after transfection of oe-Bcl2 or oe-Bcl2 + sh-Myc determined by Western blot analysis. D, Expression of PARP, Cleaved PARP, caspase 3 and Cleaved caspase 3 in cells after transfection of oe-NRMT or oe-NRMT + sh-Bcl2 determined by Western blot analysis.

**Table S1** RT-qPCR primers

| Gene | Primer sequence |
| --- | --- |
| NRMT | F: 5'-CTCCCCGATGAGATCTACCA-3' |
|  | R: 5'-TGAAGAAGAGCCCGCATAAT-3' |
| CENPA | F: 5'-GATTCTGCGATGCTGTCTGG-3' |
|  | R: 5'-CAGGCCTTTGGAACGGTGTT-3' |
| Myc | F: 5'-TGGTGCTCCATGAGGAGACA-3' |
|  | R: 5'-GTGTTTCAACTGTTCTCGTC-3' |
| Bcl2 | F: 5'-CCCTGTGGATGACTGAGTACCTG-3' |
|  | R: 5'-CCAGCCTCCGTTATCCTGG-3' |
| GAPDH | F: 5'-CCACTCCTCCACCTTTGAC-3' |
|  | R: 5'-ACCCTGTTGCTGTAGCCA-3' |

Note: RT-qPCR, reverse transcription quantitative polymerase chain reaction; F, forward; R, reverse; NRMT, N-terminal regulator of chromatin condensation 1 methyltransferase; CENPA, centromeric histone H3 variant centromere protein A; Bcl2, B cell lymphoma-2; GAPDH, glyceraldehyde-3-phosphate dehydrogenase.
